# Report from the Biennial Scientific Meeting of the Australasian Section of the American Oil Chemists Society (AAOCS) Held in Adelaide, November 2011

**DOI:** 10.3390/nu4050372

**Published:** 2012-05-23

**Authors:** Matthew R. Miller

**Affiliations:** *Secretary of AAOCS*, NZ Institute for Plant & Food Research Ltd., PO Box 5114, Nelson, New Zealand; Email: matt.miller@plantandfood.co.nz

## 1. Overview

The Australasian section of the American Oil Chemists Society (AAOCS) held their biennial meeting in Adelaide, Australia on 8–11 November 2011. Over 70 scientists, researchers and industry representatives gathered for three days of talks and discussions on lipid related topics. A highlight was the hot topic symposium on the new olive oil standard being introduced in Australia. Paul Miller, Australian Olives Association, gave a compelling address on why the standard was needed. He demonstrated that the increase in price and demand for high quality olive oils has led to products falsely or misleadingly labelled. Furthermore, the genetic and seasonal variation in minor components of olive oil has led to misclassifications. An extensive scientific and political process in Australia and overseas led to development of this new standard. Dr. Leandro Ravetti, Mordern Olives, demonstrated the development of two new methods, for analysis of pyropheophytins and diacylglycerols, are good indicators of modification by deodorisation of oils and show excellent correlation with organoleptic assessment with aging/degradation of extra virgin olive oils. Professor Rod Mailer finished this session with studies of actual adulteration cases in Australia and overseas, further highlighting the need for this new standard. 

Professor Eric Decker, University of Massachusetts (UMass), led a one and a half day workshop on lipid oxidation and antioxidants. This well received workshop highlighted the large amount of oxidation research from the Food Science department at UMass. The concept of interfacial surfaces being a key area of lipid oxidation was explored for possible oxidation prevention strategies. Eric also gave the Section opening keynote address which examined lipid oxidation in bulk oils, in particular the role of microstructures in oil. 

A two day workshop on production of margarines, dairy blends and spreads was well received. This “hands on” workshop was presented by industry leaders: Pernille Gerstenberg Kirkeby, SPX; Brad Forrest, Dansico and Lucky Inturrisi, Cargill and was led by Rod Smith, Commonwealth Scientific and Industrial Research Organisation (CSIRO) Food and Nutritional Sciences (FNS). The delegates made a series of spreads and shortenings in pilot plant facilities at CSIRO FNS, Werribee. 

Nutrition and health of fats and oils was another conference highlight. Professor Peter McLennan, University of Wollongong, described a compelling case to revisit long-chain omega 3 recommendations to fit with the evidence. In particular, evidence that fish oil reduces heart disease due to incorporation of omega-3 PUFA into the heart, independent of their effects on blood lipids or blood pressure, in low dietary concentrations. Therefore lower amounts than the recommended 500 mg/day by the National Health and Medical Research Council (NHMRC) could deliver high benefit. This message is still not getting through to consumers with medium intake under 60 mg/day in Australia. 

The biotechnology industry is growing in Australasia which was reflected by interesting presentations. Professor Colin Barrow, Deakin University, introduced his work since becoming chair of biotechnology three years ago. His presentation covered the use of lipases for concentration of omega-3 fats, stabilization and delivery technologies required for omega-3 functional foods, and development of concentration and fermentation technologies for controlled production of eicosapentaenoic acid (EPA), docosahexaenoic acid (DHA), and the related endogenous signaling molecules, resolvin E1 and neuroprotectin D1. Dr. Surinder Singh updated on the ground breaking work coming from the CSIRO Food Futures Flagship. He demonstrated the significant relative levels of EPA and DHA in genetically modified oilseeds and how these plant oils will be able to meet growing demand for these health-benefitting long-chain omega 3 oils. This impressive work recently won the CSIRO Research Achievement Medal and has achieved substantial levels of long-chain omega 3 in a series of different plants. 

A focus of the Section is to develop future researchers and industry leaders. Student presentations of orals and posters were of high quality. Will Bignell, CSIRO/University of Tasmania, presented on boosting the content of EPA and DHA in lamb towards significant dietary “source”, and won the inaugural Bryce Bell student prize for best oral. Even through drought, Will’s work in enriching sheep with omega 3 impressed the judges with crossover of traditional selective breeding techniques with genetic markers and lipid analysis. The Rod Mailer student poster prize was awarded to Kim Jye Lee Chang, CSIRO/University of Tasmania. Kim’s work screened a new collection of novel heterotrophic protists (thraustochytrids) for production of biodiesel and long-chain omega-3 oils. The Shimadzu encouragement award was won by Ramez Alhazzaa, CSIRO/University of Tasmania, for his work looking at omega 3 production in the farmed Australian fish species barramundi, *Lates calcarifer.*

## 2. Presentations

### 2.1. Why We Need a New Olive Oil Standard—A Local Solution to a Global Problem—*Paul Miller*

Olive oil has an enviable history as a food-oil and an almost peerless reputation for combined culinary and health giving properties. Olive trees and olive products make up an intrinsic part of the culture of the Mediterranean societies past and present and modern studies continually uncover new confirmations of the reason it is part of what are now touted as great diets. Olive oil’s progress to be produced in new countries, sold in new markets and used in diverse cuisines is an exciting development for human culture.

So what is the problem? 

As this product has gained favor and market demand it has commanded a well deserved premium price over other fats and oils. This premium is actually necessary because it is the only mainstream vegetable oil derived from fruits on trees—so far a more expensive business than harvesting seeds from broad-acre fields. 

The premium for olive oil and the recent increased demand for the most natural type of olive oil—extra virgin—have led to a plethora of ways both “legal” and illegal to sell products falsely or misleadingly labelled as the real thing. At the same time the spread of olive oil’s production means that its chemical makeup may vary from what is (wrongly) seen as a traditional olive oil makeup.

This presentation will briefly cover the issues confronting the olive oil sector, look at international efforts to address these issues, then detail the extensive scientific and political process in Australia and overseas that has led to the development of AS 5264-2011, the Australian Standard for Olive Oil and Olive Pomace Oils. It will also report on progress with the implementation of the Standard and offer some comments for others seeking to follow a similar path. 

### 2.2. Australian Standard for Olive Oil—World’s First Consumer Oriented Olive Oil Standard—*Leandro M. Ravetti*

Until July 2011, Australia did not have any existing Standard for olive oils. The most widely accepted international standards for olive oils are: Codex Standard and International Olive Council Standard. Other relevant standards are: European Commission Regulation and the United States Standards.

While the Australian Standard has a number of areas in common with them, the new Australian Standard differs in a number of critical aspects such as:

- Simpler and clearer commercial denomination of the different categories of olive oils.
- Establishment of best before date guidelines.
- Review of the range limits for chemical parameters in order to avoid genuine olive oil being excluded for its natural variation in composition.
- Introduction of new analytical methods which are capable of detecting modern refining techniques.

The review of chemical parameters defining olive oil considered that the current levels of fatty acids and sterols utilised in most international standards represent unnecessary obstacles to trade. The proposed changes were based on two comprehensive studies of Australian and New Zealand olive oils consisting of more than 1850 samples of oils and more than 30,000 chemical tests covering a wide range of growing conditions and varieties over a period of at least 4 years. 

Extra virgin olive oils (EVOO) are obtained from healthy olives using mechanical extraction processes that produce minimal changes in oil composition. Nowadays, it is possible to refine poor quality oils to obtain deodorised oils that are undetectable by the quality parameters included in the current IOC or Codex regulations. Australian and international research have demonstrated that Pyropheophytins (PPPs) and Diacylglycerols (DAGs) tests are good indicators of deodorised oils as well as showing excellent correlation with organoleptic assessment of oils and behaving as an accurate test to measure aging/degradation of non-deodorised extra virgin olive oils without being discriminatory in nature. 

### 2.3. The Effect of Storage Conditions on the Quality of Australian Olive Oil—*Jamie, G. Ayton, Rodney J. Mailer, Kerrie G. Graham*

The production of olive oil in Australia has increased in the last decade, with production at about 15,000 tonnes of oil last year. The majority of the oil produced was classified as extra virgin olive oil. However the conditions in which olive oil is stored can have an effect on the quality of the oil when it reaches the consumer.

This study evaluated the effect of different storage conditions on a number of olive oils produced in Australia. Samples were exposed to different temperatures, light and oxygen, and tested at intervals over a 3 year period to determine the effect these conditions had on the quality of the oil.

Total polyphenols decreased in oils exposed to oxygen and to high storage temperature. As polyphenols are powerful antioxidants, the Rancimat induction time, or shelf life, also decreased significantly over time. Alpha-tocopherol (vitamin E) was found to be significantly affected by exposure to light. Oils with less resistance to oxidation, such as those with low polyphenol content and high polyunsaturated fatty acid contents, exceeded the International Olive Council (IOC) limit for peroxide value (20 mEq O_2_/kg oil) within 8 months when exposed to oxygen. As a result of this, the same oils exceeded the requirements of the IOC for UV absorbance (K232 and K268—measures of primary and secondary oxidation in oil), after only 6 months storage. 

The initial composition of the oil had a significant bearing on the quality of olive oil when exposed to different storage conditions. This study shows the importance of storage conditions in maintaining the quality of extra virgin olive oil. This has major implications in the olive oil industry in maintaining adequate storage conditions from processing through to bottling and marketing in retaining a premium product.

### 2.4. Phenolic Profile and Sensory Attributes of New Zealand “Frantoio” Olive Oil—Influence of Maturity—*Marie Wong, Jenkins Ogwaro, Cecilia Requejo-Jackman, Miriam Farrell, Shane Olsson, Michelle Beresford, Yi Wang, Margret Edwards, Mark Wohler, Allan Woolf*

Extra virgin olive oils produced in New Zealand have been found to have distinctive composition and flavor characteristics. The objective of this research was to identify the impact of growing region and harvest maturity on the phenolic composition and sensory attributes of oil extracted from “Frantoio” olives. Olives were harvested at different stages of maturity, after full bloom, from orchards in three regions (Hawkes Bay, Bombay and Waiheke Island). The olives were assessed for a variety of maturity indices and oil was extracted either by solvent or by cold pressed (CP) extraction. The phenolic composition of the oils was determined by HPLC and the CP oils were evaluated by a trained sensory panel. The % oil (by dry weight) generally increased with maturity up to about 185 days after full bloom, beyond which the % oil did not increase; regional differences in % oil were observed. As expected total phenolics were found to decrease with maturity and were found to be strongly correlated to the sensory bitterness, pungency and overall acceptability of the oils. Results from a chemical test for intensity of bitterness (K_225_) were found to be correlated to the sensory bitterness scores. Several simple phenols were quantified in the oils and the simple phenols hydroxytyrosol and tyrosol declined with fruit maturity. Luteolin was the main flavonoid identified. The major phenolic compounds present in the oils were found to be the secoiridoids; significant differences in individual secoiridoids were found between the orchards. Strong correlations between sensory bitterness and concentration of individual secoiridoids demonstrated the important role played by these compounds in the flavor of virgin olive oil. The dominant flavors and aromas in the oils were “bitter salad”, “fresh green bean”, “vanilla toffee”, “walnut” and “black pepper”. This study revealed that the phenolic content and sensory attributes of the oils were influenced by orchard location and harvest maturity. 

### 2.5. Maturity Indices for New Zealand Olive Growers—*Cecilia Requejo-Jackman, Marie Wong, Jenkins Ogwaro, Miriam Farrell, Shane Olsson, Michelle Beresford, Roger Harker, Yi Wang, Mark Wohler, Allan Woolf*

Olives are grown in 12 distinct regions in New Zealand (NZ). NZ’s maritime environment is very different to the Mediterranean environment where much of the traditional olive growing and harvesting information is derived. NZ olive growers estimate the maturity of their fruit before harvest with a variety of qualitative methods. Measures used in the northern hemispheres such as maturity index (MI—a measure of external and internal colour) have not been found to be a reliable tool in NZ. Hence, the objective of this research was to identify qualitative or quantitative measures of maturity for olives grown in NZ. A three year study was undertaken in NZ. Fruit from six cultivars (“Frantoio”, “Leccino”, “Koroneiki”, “Barnea”, “Picual”, and “J5”) were sampled from over 20 orchards throughout NZ from as early as February through to July. This presentation will summarise key findings from this three-year study. Harvested olives were assessed for a number of maturity indices (% oil content, MI, % dry matter (% DM), total solids (DM/fruit), firmness and fruit weight). Oil was extracted from the fruit by solvent extraction for % oil content and compositional information, and by cold pressed (CP) extraction for sensory evaluation and storage. Throughout the harvest season oils were analyzed to follow changes in total phenolic content. Solvent extracted oils were also analyzed to determine the variation in fatty acids, tocopherol and sterol composition from various cultivars and regions in NZ. The % oil content (on a dry weight basis) increases with maturity. MI and % DM were not good predictors of oil content. However, total solids and firmness showed strong correlations with % oil content. Regional and cultivar differences in % oil, fatty acid composition and tocopherol content were observed. Cold pressed extra virgin olive oils were evaluated for their sensory attributes by a trained sensory panel before and after long-term storage. Achieving maximum oil yields while producing well balanced oils with fruity, bitter and pungent flavor characteristics is still a major challenge for the olive grower under NZ’s relatively wet growing environments. 

### 2.6. Authenticity and Adulteration of Olive Oils—*Rodney J. Mailer*

Australia is a net importer of olive oil with over 30,000 tonnes of oil imported annually. The oil comes mainly from Spain, Italy and Greece, either bottled or in bulk for bottling after arrival. In most cases the oil has been purchased with very little testing to ensure authenticity. The integrity of the product has been left to the scrutiny of the International Olive Council (IOC) which receives representative samples from around the world each year for testing. The samples from Australia are provided to the IOC by the Australian Olive Oil Association (AOOA). Despite this ongoing screening, there are no records of Australian imports being found to be out of specification or having been rejected. 

In recent years Australia has developed two internationally accredited olive oil laboratories which now regularly test both imported and domestic products to ensure that the oil meets the specifications for the grade and that it is free from adulteration. Subsequently, many oils have been sampled from supermarket shelves and analyzed by the Australian labs. The methods used are often complex, time consuming and expensive to carry out but provide clear evidence of the authenticity of the oil. Alarmingly, a large number of the oils fail the IOC tests. Many fail because they are old or have been stored poorly. But some have clearly been adulterated with seed oils such as canola or refined olive oil including pomace oil. The sale of these products as extra virgin olive oil constitutes fraud and the Australian consumers are not getting what they pay for. 

Australia has been restricted by the lack of an Australian standard to encourage authorities to take action on fraud despite the availability of reliable data. The new Australian standards now accurately describe the methodology and the acceptable limits for olive oil in Australia. The methods used to determine authenticity and some of the recent investigations will be discussed.

### 2.7. Olive Oil—More than Monounsaturated Fat—*Rosemary A. Stanton*

Around the Mediterranean, people have been enjoying olive oil for thousands of years. Its popularity stems from its ready availability throughout the region, but also because the flavor of fruity olive oil is an integral part of the many variations of Mediterranean diets.

When Ancel Keys and his co-workers conducted their famous Seven Countries Study in the 1950s, they assumed much of the protection from cardiovascular disease in Crete and Southern Italy was due to the unsaturated fatty acids in olive oil. Many studies have since shown the heart health value of unsaturated fatty acids, but dwelling on this alone underestimates the true value of olive oil.

Switching from a diet high in saturated fats to one with more unsaturated fats lowers LDL cholesterol. Any vegetable oil will achieve this, and indeed, oils dominated by polyunsaturated fatty acids may have a greater effect than those rich in monounsaturated fatty acids, such as olive oil. The real message, however, is to consider whole foods rather than isolated nutrients. And this is where extra virgin olive oil comes into its own and gains the edge on other oils.

As well as its unsaturated fatty acids, extra virgin olive oil contains more than 30 compounds, including polyphenols such as hydroxytyrosol, oleuropein and oleocanthal. These compounds play a role in preventing the oxidation of LDL cholesterol, controlling blood pressure and platelet function and moderating anti-inflammatory responses within the body. This latter function may be the most important reason to make extra virgin olive oil the oil of choice, especially for protecting against the metabolic syndrome. Components in extra virgin olive oil may also interact with genetic factors in providing protection against breast and other common cancers and Alzheimer’s disease. Research continues.

It is always difficult to isolate the benefits of any single food in the diet. Most nutrition research starts from an epidemiological perspective (usually long-term) and progresses to studies of cohorts and short-term, randomised controlled trials of some aspect of the total diet. In real life, however, the real test for any nutritional intervention or advice revolves around factors such as taste, price and convenience. For taste, extra virgin olive oil scores another win. Perhaps the ultimate beauty is that the same factors that provide the flavor in extra virgin olive oil also provide many of its health benefits. Good taste and health make an ideal partnership.

### 2.8. Re-Examining the Mechanisms of Lipid Oxidation in Bulk Oils, the Role of Physical Structures—*Eric A. Decker, Bingcan Chen, David J. McClements*

Lipid oxidation in bulk oils is highly dependent on minor components in the oils. These minor components have traditionally been postulated to impact oxidation rates through properties such as metal chelation and decomposition of lipid hydroperoxides. However, another potential mechanism by which minor components can impact lipid oxidation in bulk oils is through their ability to form physical structures in the presence of small amounts of water. These nanostructures which include reverse micelles and lamellar bilayers are known as association colloids. Techniques such as interfacial tension and small angle X-ray scattering have been used to show that the combination of phospholipids and water will result in the formation reverse micelles in bulk oils. These reverse micelles increase lipid oxidation rates in a manner that is independent of the chemical properties of the phospholipids. Phospholipid reverse micelles in bulk oils can also interact with antioxidants such as tocopherols and Trolox. These interactions can concentrate the antioxidants at the lipid-water interface, a process that can increase the activity of the antioxidants. However, when antioxidants concentrations are increased the physical structures can allow the antioxidant to interact with metals leading to an acceleration of oxidation. The role of association colloids in lipid oxidation mechanisms in bulk oils is very complex. However, understanding how these physical structures impact the activity of both prooxidants and antioxidants could provide new insights into innovative technologies to control lipid oxidation.

### 2.9. The Edible Oils and Fats Industry: More than a Source of Grease—*Mahinda Y. Abeywardena*

The world consumption of edible oils and fats in recent years has consistently been rising. The global production of vegetable oils during 2008/09 has been estimated at 133 million tons, the forecast for 2010/11 stands at 145 million tons, an increase of almost 10% over the previous year. Similarly, the demand for phytochemicals (natural antioxidants and related plant-based bioactives) is increasing steadily as a result of a growing scientific body of evidence which has also paved their entry into different market segments, including the food, supplement and nutraceutical sectors. The vegetable oil industry is a major source of natural antioxidants including tocopherols, tocotrienols and carotenes. Similarly, plant sterols and stanols used in functional foods as cholesterol-lowering ingredients are extracted from the deodoriser distillates of vegetable oil refining (e.g. soybean, corn), and also from tall oil (a by-product of paper pulping industry). More recently, waste streams have been identified as potential sources of bioactives with leaves from the olive tree (*Olea oleuropaea*), olive pomace, olive mill waste-water, oil palm fronds *(Elaeis guineensis)* and palm oil mill waste being confirmed as rich sources of polyphenols. Compared to the importance of vitamin antioxidants (E and C) and carotenoids, which have been known for sometime, the potential benefits of dietary polyphenols have emerged relatively recently, and indicate that plant polyphenols possess a range of health benefits that extend beyond their antioxidant/radical scavenging activity. Indeed, polyphenols possess multi-functional properties within the same molecule, favouring pleiotropism. In the present study we evaluated a novel bioactive preparation enriched in polyphenols (oil palm phenolics; OPP), recovered from the large aqueous biowaste generated during the milling process and extraction of palm oil. Numerous health benefits associated with OPP have been identified, and further investigations are underway. 

### 2.10. The Health Implications of Lower Fat, Higher Carbohydrate Diets: Macronutrient Exchanges, Macronutrient Quality and Nutritional Adequacy—*Bill S. Shrapnel*

The Seven Countries Study generated the Asian model for coronary heart disease prevention—a high carbohydrate diet, low in total and saturated fat. The efficacy of this model in modern western societies is now in question following the publication of a pooled analysis of prospective cohort studies suggesting that dietary saturated fat and carbohydrate confer similar coronary heart disease risk. If true, the coronary risk associated with higher carbohydrate, lower fat diets will not be determined by the replacement of saturated fat by carbohydrate, but by the other macronutrient exchanges that effectively take place.

The aim of this study was to explore the relationships between dietary macronutrients, macronutrient quality and nutritional adequacy in the diets of free-living Australian teenagers and to consider the implications for total macronutrient recommendations. Using the Children’s Nutrition and Physical Activity Survey (2007) database, the nutrient intakes of boys and girls aged 14–16 years were assessed by quintile of carbohydrate intake.

Carbohydrate intake was inversely associated with intakes of total fat, saturated fat, polyunsaturated fat, long-chain omega 3, monounsaturated fat and protein. Fat quality (P/S ratio) was constant and carbohydrate quality declined across quintiles of carbohydrate intake. Nutritional adequacy was generally compatible with a wide range of fat and carbohydrate intakes. 

In combination, the effective exchanges of carbohydrate for polyunsaturated fat, long-chain omega 3, monounsaturated fat and protein as total carbohydrate intakes increased would be expected to increase coronary disease risk via several mechanisms. This implies that there may be a progressive increase in coronary risk as carbohydrate intakes increase across the Acceptable Macronutrient Distribution Range (AMDR). There may be a case for lowering the upper and lower boundaries of the AMDR for carbohydrate intake and increasing and the upper and lower boundaries of the AMDR for fat as a strategy for chronic disease prevention. 

### 2.11. Getting to the Heart of Omega-3 Fatty Acid Health Benefits: Reconciling the Recommendations with the Evidence—*Peter L. McLennan*

Health authorities globally recognize, for heart health, the importance if not the essentiality, of long chain *n*-3 PUFA and the foods that contain them. There is however little understanding of the mechanisms and only patchy clinical uptake. 

In Australia, the National Heart Foundation recommends regular intake of *n*-3 PUFA by healthy adults (500 mg·day^−1^ EPA and DHA), yet there is a focus on potential mechanisms that are only achieved by daily intakes of 1000–4000 mg or more. These include triglyceride lowering, modest blood pressure lowering, reduced vascular stiffness and anti-inflammatory effects, for which there is level I and II evidence. This reflects a slavish adherence to the classical diet-heart hypothesis as the basis for *n*-3 PUFA effects, and focus on classical risk factors for atherosclerosis and coronary artery disease. These factors typically require therapeutic doses higher than can be usually obtained in the diet; well above intakes of 1–2 fish meals per week or fatty acid equivalents identified in prospective cohort studies, case control studies and some randomized clinical trials. The recommended target intake is therefore a compromise between epidemiology and therapeutics and is estimated from the 90th centile of usual population intake, rather than defined clinical endpoints. 

Collectively, the epidemiology, intervention trials and nutritional physiology research compel us to consider a new paradigm of nutritional preconditioning based on mechanisms other than modulation of classical risk factors. This includes heart rate lowering, prevention of sudden arrhythmic death, reduced risk of incident heart failure and progression of heart failure, and is better attributed to the incorporation of dietary n-3 PUFA into myocardial membranes than to prevention of coronary artery disease. Clinical uptake of nutritional recommendations and application of guidelines will increase only when they are achievable and describe clearly understood mechanisms based on evidence.

### 2.12. Erythrocyte Omega-3 Levels—An Alternative Basis for Intake Recommendations—*Peter R. C. Howe*

As public awareness of the health benefits of fish oil supplementation continues to escalate, there is increasing demand for advice from health professionals on intake requirements for specific indications. However, both the requirements and the extent of potential benefit will be influenced by an individual’s omega-3 status, which is determined by habitual dietary omega-3 consumption. The Omega-3 Index, a measure of the long chain omega-3 fatty acid content of red blood cells, provides a simple clinical measure of an individual’s omega-3 status; it also serves as a new risk factor for cardiovascular disease, particularly sudden death, and possibly other health risks. The limited data available suggest that the median omega-3 status of Australian adults is well below optimal levels for heart health and may also be associated with poorer metabolic and mental health. Population intake recommendations, which are based on median omega-3 intakes, should instead be based on intakes required to attain optimal omega-3 status. Moreover, regular monitoring of omega-3 status should also be used to guide omega-3 dose requirements in treating a wide of range of health conditions, as either sole or adjunct therapy. Thus the Omega-3 Index is an important new tool in the management of chronic conditions.

### 2.13. Omega-3s, Cognition and Mood in Older Australians with Memory Problems—*Catherine M. Milte*

Suboptimal Omega-3 (*n*-3) polyunsaturated fatty acid (PUFA) status assessed through erythrocyte PUFA levels may contribute to both depression and dementia. Depressive symptoms may contribute to an increased rate of progression to more severe forms of dementia. Consumption of the *n*-3 PUFAs, eicosapentaenoic acid (EPA) and docosahexaenoic acid (DHA), and resulting increase in *n-*3 PUFA status may alleviate some of the cognitive and depressive symptoms associated with mild cognitive impairment (MCI).

Erythrocyte PUFA levels (% of total), memory, cognition and mood were investigated in 50 adults ≥65 years with MCI and 29 healthy aged-matched controls. A 6-month randomised controlled parallel trial was conducted to determine the effects of supplementation with EPA-rich oil, DHA-rich oil or linoleic acid (LA)-rich safflower oil on memory and depressive symptoms in the MCI group.

At baseline, erythrocyte EPA was lower in the adults with MCI compared with the controls (0.94% *vs.* 1.26%, *p* < 0.01), whilst Omega-6 (*n*-6) PUFAs were higher: dihomo-gamma-linolenic acid (1.51% *vs*. 1.32%, *p* < 0.01), arachidonic acid (11.54% *vs*. 10.70%, *p* < 0.01), *n*-6 docosapentaenoic acid (0.46% *vs*. 0.34%, *p* < 0.01) and total *n*-6 PUFA (24.12% *vs*. 23.37%, *p* < 0.05). Adults with MCI also had higher depression scores than healthy controls at baseline (3.05 *vs*. 1.33, *p* < 0.01). In the MCI group at 6 months, DHA supplementation improved verbal fluency (*p <* 0.05), and both EPA and DHA supplementation improved depression scores compared with LA supplementation (EPA: *p* < 0.05, DHA: *p* < 0.01). Improvements in depressive symptoms were associated with increased erythrocyte DHA + EPA (*r* = 0.39, *p* < 0.05) over 6 months.

Increasing *n*-3 PUFA status by increased intake of EPA and DHA may improve mood and cognition in older adults with MCI. The effect of these improvements on dementia risk and progression needs to be investigated further.

### 2.14. Effect of Saturated Fats on Nutrition and Health: An Update—*Kalyana Sundram*

Dietary fat recommendations traditionally advocated low fat and low-saturated fat diets, perceived to be beneficial for cardiovascular health. Recommendations focused largely on fat quantity and composition. However, during the past decade these engrained recommendations have been challenged and there is a new and emerging paradigm shift in the way we perceive saturated fats. Much of this was a realization that trans fat negatively impacts human plasma lipoproteins (increased TC, LDL-C, Lp (a) and decreased HDL-C relative to their *cis isomers*) and have untoward implications for cardiovascular disease risk. Through meta-anlaysis, clinical and epidemiological data, saturated fats are now been shown to potentially have a less adverse impact on disease outcomes compared to the original hypotehesis that resulted in strategies towards their avoidance. Added to this is the emerging debate that all saturated fatty acids are not equal in their cholesterolaemic effects with some scientific evidence (although not conclusive) for the neutrality of stearic acid. Less certain are the comparative hypercholesterolaemic behaviour of different oils and fats that are termed saturated, yet contain significant amounts of both monounsaturated and polyunsaturated fatty acids. Despite such emerging evidence, we remain unsure about how consumers should be correctly advised and these issues are discussed in the presentation. 

### 2.15. Estimation of Individual Classes and Total Antioxidant Intake of Australians—*Katie M.M. Bauer, Janet Bryan, Karen J. Murphy*

Antioxidant (AOX) consumption has reported health benefits such as reducing cardiovascular disease risk factors, improving endothelial function and delaying age-related cognitive decline. However there is little information available on the dietary intake of total and individual classes of AOXs of Australians. 

To estimate the total dietary AOX intake of 1183 South Australians in a cross sectional study. 

Pre-existing data collected through electoral roles from 1183 middle aged (51 ± 6 year) men and women was used to estimate total antioxidant intake. AOX intake including vitamin A, C, E, carotenoids, flavonoids, isoflavones and selenium was estimated using a validated FFQ. Reported intakes were converted to serves per day using serving sizes described for relevant foods in Foodworks Professional (Xyris). AOX content of foods was determined using a combination of Foodworks Professional and the USDA Antioxidant Databases (USDA 1998, 2004, 2007, 2008). One-way ANOVA was undertaken to examine differences between genders for AOX intake (SPSS V17.0).

Estimated total antioxidant intake (sum of vitamin A, C, E, selenium, carotenoids, flavonoids and isoflavones) was 831 ± 597 mg/day. Men and women consumed 766 ± 578 mg and 868 ± 605 mg of total AOX per day, respectively. Flavonoids was the major AOX consumed (623 ± 579 mg/day) of which thearubigin, was the major polyphenol (364 ± 395 mg/day). There was no difference between gender for the consumption of vit A, C or isoflavones, however men consumed more vit E, and selenium (*P* < 0.0001) than women and women consumed more carotenoids, total flavonoids and thearubigins (*P* < 0.01) than men. Of the flavonoid class, men consumed more flavanones than women (*P* = 0.01) whereas women consumed more anthocyanidins (*P* < 0.0001), flavan-3-ols, flavones and flavonols (all *P* < 0.05).

Gender differences in AOX intakes appear to reflect difference in dietary habits. These data indicate that flavonoids, mainly from black tea, are the main AOX consumed by Australians. To our knowledge this is the first detailed study to estimate intakes of total AOX and individual classes of AOX for men and women.

### 2.16. Production of a Low Fat Dairy Spread Based on a Natural Fat Replacer—*Pernille Gerstenberg Kirkeby*

Many consumers today are health conscious and willing to pay a little extra for high quality products. This group of consumers is not only focusing on controlling their fat intake but is also requesting products which are compounded of natural ingredients. It is therefore of interest to manufacture a high quality dairy spread with a fat content of 40% stabilized by microparticulated whey. Microparticulated whey can be considered as a natural ingredient but is a relatively costly stabilizer; however, it offers great functionality as fat replacer such as improved mouth feel and minimized instability during spreading. 

Using a standard SPX pilot plant designed to crystallize various fat products, 40% dairy spread samples were produced by applying different processing conditions during the trials. All processing parameters as well as the results of the individual sample evaluation will be presented and discussed. All samples were evaluated in relation to a standard full fat dairy spread.

### 2.17. Extraction and Fractionation of High Value Lipids Using Supercritical Fluids—*Owen J. Catchpole, Stephen J. Tallon, Peter J. Dyer, Andrew D. Mackenzie, Mikhail V. Vyssotski, Fernando Montañés*

This presentation reviews recent work carried out by IRL on the extraction and fractionation of high value lipids using supercritical fluids from a variety of biomasses. The high value lipids include complex lipids from marine organisms and biomass from fermentation processes; and highly concentrated long-chain polyunsaturated fatty acids (LC-PUFA). Supercritical CO_2_ has some significant limitations regarding the extraction of high value phospholipids and glycolipids. To overcome those limitations, we have used both CO_2_ with ethanol as a co-solvent for dry biomasses containing these lipids; and dimethylether for both wet and dry biomasses. The extraction of phospholipids rich in phosphatidylcholine and LC-PUFA can be achieved using CO_2_ + ethanol, and results are presented for selected marine biomasses. CO_2_ + ethanol is a poor solvent for phosphatidylserine and phosphatidylinositol and these remain behind in the biomass. In contrast, DME is a non-selective solvent for phospho- and glycolipids, and these can even be extracted from wet biomass. Results are presented for the extraction of lipids from fermentation biomass using DME. Supercritical CO_2_ can be incorporated with conventional processing technologies to concentrate LC-PUFA in the form of fatty acids or ethyl esters. Technologies using CO_2_ and urea fractionation, enzymatic esterification, and chromatography are presented, which in combination can enable the production of single long-chain polyunsaturated fatty acids in high concentration.

### 2.18. Adsorbent Purification of Frying Oils—*Brian S. Cooke*

Regardless of origin, virgin frying oils are non-polar in nature. However, polar compounds are formed as these oils degrade due to oxidation, hydrolysis, heat stress, etc. It is generally accepted that polar compounds are a reliable measure of oil quality and correlate to the taste, appearance and nutritional properties of fried foods. The concentration of polar materials increases until the oil becomes unfit for use. Countries have established legal limits or guidelines for polar materials, polymers, and free fatty acids in the oil. 

Synthetic magnesium silicate has been shown to have a strong adsorptive affinity for polar materials. The effects of daily treatment of frying oil with DALSORB®, a magnesium silicate-based adsorbent, were observed in a commercial frying operation. The use of DALSORB® allowed for continued frying operation with no oil discard by sharply reducing levels of free fatty acid (91%), alkaline materials (86%), colors (70%), total polar compounds (47%), polymers (20%) and anisidine value (20%).

### 2.19. A Review of Rice Bran Oil Technology and Comparison of Market Samples—*Geoff Webster*

A quick review of production volumes, processing, nutrition and utilisation will provide insight into this relatively niche oil. The variation of reported processing methods and processing aids will be examined to try to establish which processes and aids give the best quality oil. A selection of Australasian market rice bran oils will be analyzed to compare their freshness, nutritional value, their frying life and shelf life.

### 2.20. Use of Combined Chromatographic Techniques for Characterisation of Long-Chain Omega-3 Oils—*Maged P. Mansour, Peter A Fagan, Chakra Wijesundera, Zhiping Shen, Peter D. Nichols*

Considerable evidence exists that omega-3 long-chain polyunsaturated fatty acids (ω3 LC-PUFA, also termed LC omega-3 oils) are important in the prevention and treatment of a range of human diseases and in the development of neurological functions in infants. Since these highly unsaturated ω3 LC-PUFA can be rapidly oxidised, various delivery systems have been developed to allow their incorporation into food products. Naturally occurring ω3 LC-PUFA are predominantly found in marine sources (selected bacteria, algae and fish), with high concentrations occurring in oily fish species (e.g. tuna, salmon, bonito) through bioaccumulation up the food-chain. Increased pressure on wild fish stocks has prompted extensive global research into generating new sources of these LC omega-3 oils. CSIRO has been actively investigating LC omega-3 oils for many years in areas including the more recent focus on the development of new sources of LC omega-3 oils from terrestrial land plants, characterization of novel marine oils for enhanced utilization of fisheries by-products, microencapsulation of LC omega-3 oils to improve oxidative stability and bioavailability of LC omega-3 oils. We provide a general overview of chromatographic techniques and analytical methodologies developed, refined and employed by CSIRO in a range of omega-3 oil activities. Techniques include: (i) Rapid and 2D-GC methods for the analysis of ω3 LC-PUFA; (ii) Head space sampling techniques coupled with GC-FID/MS for monitoring oxidation of important ω3 LC-PUFA, such as DHA [docosahexaenoic acid, 22:6(ω3)]; (iii) Effect of regiospecificity on oxidative stability and bio-availability of LC omega-3 oils; (iv) Argentation techniques for isolation and separation of PUFA; (iv) 4,4-dimethyloxazoline (DMOX) derivatives for MS analysis of novel fatty acids and (v) TLC-FID, HPLC and LC-MS techniques to assess lipid class pathway changes including in GM land plant derived oils.

### 2.21. Signature Lipids—Where are We at and What is Next?—*Peter D Nichols, Heidi Pethybridge, Patti Virtue, Katrina Phillips, Kátya Abrantes, Jeff Drazen, Jock Young, Rick Phleger*

The application of signature lipid methodology is being increasingly demonstrated and applied for a range of fish, squid and higher predators, as well as in environmental studies. In food web studies, the signature lipid approach complements traditional stomach content methodologies, and generally can provide a longer term dietary signal, as well as information on fish condition. The approach is particularly valuable where sample sizes are small, or when conventional biological techniques such as stomach content analysis can not be performed, for example with live marine mammals. In the latter case, biopsy samples can be obtained and analyzed. Protocols include lipid extraction and class profiling, and fatty acid methylation followed by gas chromatography (GC) and GC-mass spectrometry (GC-MS) analyses. Effort has occurred to increase sample through-put to the levels required in modern biological studies. 

Case studies highlight the application of the signature lipid approach to a range of species, with for example digestive gland fatty acid profiles of squid indicating that these species were reliant on a myctophid-based diet. The deep sea rattail was shown to be feeding on squid carrion rather than on benthic fauna as previously proposed. It is also possible to examine the effect of regional, temporal and other differences including maturity on diet and the overall role of lipid in marine species including large predator species such as swordfish and tuna. The use of signature lipid data for the determination of the diet of lower tropic levels including zooplankton, molluscs and higher predators such as cetaceans and pinnipeds can be achieved. 

The case studies undertaken have been informative to date, and the approach is now available for integration with other research including within ecosystem models. The use of signature lipid methodology offers potential, via sampling and analysis on a broader temporal and spatial basis, to also examine key environmental influences, including climate change and other factors, on commercial and other marine species.

### 2.22. The Use of Plant and Animal Oils to Improve the Sustainable Production of Yellowtail Kingfish *Seriola lalandi—Jenna N. Bowyer, Jian G. Qin, Richard P. Smullen, Louise R. Ward, David A. J. Stone*

The most current global issue regarding the production of fish is the sustainable use of marine derived ingredients, such as fish meal and fish oil from wild fisheries, by using alternative dietary ingredients. Sustainable use of dietary fish oil is measured by the “fish in fish out” ratio (FIFO). This study investigated the feasibility of replacing fish oil with canola oil and poultry oil in diets for yellowtail kingfish (95 g) (*Seriola lalandi*; YTK); a carnivorous, marine species currently produced for aquaculture. 

The study was conducted at optimal (22 °C) and sub-optimal (18 °C) water temperatures. Diets were formulated to contain 45% crude protein and 25% crude lipid. Five diets were tested. Fish oil was added at 20%, or substituted with canola oil at 10 or 20% (FO/CO, CO), or poultry fat at 10 or 20% (FO/PO, PO). Residual FO from fish meal contributed ~5% to the total lipid content of each diet. Growth performance, feeding efficiencies, fillet fatty acid (FA) profiles and FIFO were measured. 

Weight gain was affected by temperature (22 >18°C) and diet (FO/PO ^a^, PO ^ab^, FO ^ab^, FO/CO ^b^, CO ^c^ like letters = no significant difference; *P* < 0.001), without interaction (*P* > 0.05). The effects of diet on feed conversion rate of fish fed CO at 18 > 22°C (*P* < 0.001). Fillet FAs were affected by temperature and diet (*P* < 0.05), but some FA classes were affected by an interaction (*P* < 0.05). The alternative lipids reduced essential omega 3 long chain polyunsaturated FA levels in fillets compared to FO, but fish were not market size and levels can be improved through phase-feeding. 

Results suggest complete substitution of FO with PO is possible with ~5% residual FO, but CO is not recommended. The use of alternative oils improved the FIFO and will ensure the sustainable production of YTK. 

### 2.23. Vegetable Oils in Tropical Aquaculture: towards a Sustainable Future—*Ramez R. Alhazzaa, Andrew R. Bridle, Peter D. Nichols, Chris G. Carter*

Fish oil (FO) is the major source of dietary lipid in carnivorous fish including barramundi, *Lates calcarifer*, which is widely farmed in Asia and Australia. However, recent increases in FO prices, increased demand and the foreseen inability of wild fisheries to meet future requirements have created a need for cheaper and more sustainable alternatives. Vegetable oils (VO) can be produced in sufficient quantities to meet the growing aquaculture demand, although they lack the long-chain (≥C_20_) polyunsaturated fatty acids (LC-PUFA) beneficial to human consumers. Some VO like canola oil (CO) and echium oil from *Echium plantagineum* (EO) have high levels of *n*-3 and *n*-6 short chain (≤C_18_) polyunsaturated fatty acids (PUFA) that can accumulate or converted into (LC-PUFA) by some fish species, although generally at low efficiency, and not to DHA. In a series of comparative and factorial experiments, we investigated the growth and quality of barramundi fed on different dietary oils: FO, CO and EO over a wide range of salinities, temperatures and subject to stressors. In general, growth performance parameters were comparable for FO and VO treatments, and resulted in accumulation of VO derived *n*-3 and *n*-6 PUFA. Salinity has no direct effect on barramundi growth or lipid metabolism regardless of the dietary lipid source. The growth of barramundi slows in cold (20 °C) water compared to optimal (30 °C) temperature. PUFA deposits in muscle from dietary VO are maintained under rapid temperature decreases. In contrast, excess LC-PUFA from FO were depleted faster than in VO fed fish. The production of eicosanoid metabolites in fish fed FO was higher than for fish fed VO following bacterial infection. EO-fed fish had significantly higher levels of eicosanoids than those fed CO. In summary, barramundi fed on VO are a rich source of LC-PUFA precursors, ALA (18:3*n*-3) and SDA (18:4*n*-3), and grow well under the different environmental conditions that are typical of outdoor barramundi farms. The use of terrestrial VO containing the LC-PUFA precursors show promise for use in barramundi aquafeed in terms of fish growth and health as either partial or complete alternatives for FO. However, high content of the n-3 LC-PUFA—EPA and DHA—is not achieved. 

### 2.24. Intramuscular Long-chain Omega 3 Content in Australian Lamb: Drought Effect, Genomics and Dietary Improvement Strategies—*Will C. Bignell, Peter D. Nichols, James W. Kijas, Russel McCulloch, Aduli E.O. Malau-Aduli*

Australian consumers are becoming increasingly aware of the health benefits of long-chain (≥C20) omega-3 polyunsaturated fatty acids (LC-PUFA)—eicosapentaenoic acid (EPA), 20:5ω3 and docosahexaenoic acid (DHA), 22:6ω3. Despite this increased awareness Australians are generally not consuming the NHMRC suggested daily target (500 mg/day) of EPA + DHA. This study investigated the current content of the LC-PUFA, EPA + DHA, in five commonly used sheep meat breeds and the potential to increase intramuscular LC-PUFA content through dietary manipulation and genomics. The study used 500 first cross first cross Merino lambs sired by Texel, East Friesian, Dorset, Coopworth and White Suffolk rams. The flock were raised on severely drought affected pastures with limited periods grazing irrigated pasture. At seven months of age a sub sample of 40 animals was removed for a 60 day feeding trial and 93 animals with live weights over 44.5 kg were slaughtered. The remaining flock were relocated to northern Tasmania which was not experiencing drought. Thirty-one single nucleotide polymorphisms (SNP) were genotyped in *Longissimus dorsi* muscle samples from 362 of the crossbred prime lambs sired by the five genetically divergent rams and genetic association between LC-PUFA, delta-6 desaturase (FADS2) and fatty acid binding protein (FABP4) gene clusters investigated. 

Pasture reared animals were slaughtered at 44.5 kg liveweight in three slaughter groups and feeding trial animals were slaughtered upon conclusion of the trial. Mean EPA + DHA content of animals reared solely on drought affected pasture was 7.5 ± 0.7 mg/100 g in comparison to the relocated animals which achieved 15.2 ± 0.5 mg/100 g. 60 day supplementation with canola meal or cracked lupins increased intramuscular EPA + DHA content to 12.6 ± 0.4 mg/100 g with canola and 14.2 ± 0.5 mg/100 g with lupins. These findings indicate that drought has a negative impact on the intramuscular EPA + DHA content, however, relocation and strategic supplementation can improve LC-PUFA content, but still did not reach the dietary “source” level of 30 mg/100 g. Genetic associations between the SNP markers and LC-PUFA may have a direct effect on functional lipid synthesis pathways that could potentially be markers of choice for prime lamb producers to effectively select for enhanced muscle long-chain omega-3 content in their breeding flock. When used in conjunction with dietary supplementation, it could boost lamb towards dietary “source” content of EPA + DHA, and thereby enhance Australia’s population health through consumption of a higher quality lamb meat.

### 2.25. Omega-3 Biotechnology: Functional Foods and Pharmaceuticals—*Colin J. Barrow*

The health benefits of long-chain omega-3 fatty acids are well established, especially for eicosapentaenoic acid (EPA) and docosapentaenoic acid (DHA) from fish and microbial sources. In fact, a billion dollar market exists for these compounds in nutritional supplements, functional foods and pharmaceuticals. This presentation will describe some aspects of omega-3 biotechnology important for current functional food and pharmaceutical development. These include, the use of lipases for the concentration of omega-3 fats, stabilization and delivery technologies required for omega-3 functional foods, and the development of concentration and fermentation technologies for controlled production of EPA, DHA, and the related endogenous signaling molecules, resolvin E1 and neuroprotectin D1.

### 2.26. Towards Designer Lipids: Regiospecific Analyses of NZ Marine Oils—*Matthew R. Miller, Nigel J. Perry, Elaine J. Burgess, Susan N. Marshall*

The lipid profiles of two major New Zealand marine oil sources were investigated with particular attention to their regiospecific nature using 13C-nuclear magnetic resonance (NMR) analysis. Oils from hoki, *Macruronus novaezelandiae* sp., and green lipped mussel *Perna canaliculus* (GLM) were analyzed for their lipid content, lipid class and fatty acid (FA) profile. The regiospecificic distribution of long chain (C ≥ 20) polyunsaturated fatty acids (LC-PUFA) between the *sn*-1,3 and *sn*-2 glycerol chains was established, calculated from the carbonyl region in the triacylglycerol fraction. Commercial rendered hoki oil (RHO) produced from the viscera and filling discards, had a very similar lipid profile to that of commercially-produced hoki liver oil (HLO) confirming that the liver is the major source of oil in RHO. GLM oil extracted in the laboratory using solvent extraction had a different lipid profile to a commercial mussel oil (CMO, Lyprinol^®^) extracted using supercritical CO2 technology. This is most likely due to the different extraction method and the addition of high proportion of olive oil in the CMO. The regiospecific distribution of fatty acids showed significant differences between the oils, specifically in the LC-PUFA. Docosahexaenoic acid (DHA) had a preference to the *sn-*2 position in all oils (59.2% HLO, 54.3% RHO, 63.4% GLM and 44.3% CMO). Eicosapentaenoic acid (EPA) had a more even distribution between the 3 positions on the triglyceride backbone in hoki oil (29.1% HLO, 33.6% RHO on *sn*-2) while there was a slight *sn*-2 positional preference in the GLM oils (37.6% GML, 41.1% CMO). This regiospecificic information is vital to distinguish omega 3 LC-PUFA rich marine oils from other marine sources for oil authentication purposes.

### 2.27. Biodiscovery of New Australian Thraustochytrids for Production of Biodiesel and Long-Chain Omega-3 Oils and Co-Products—*Kim Jye Lee Chang, Graeme A. Dunstan, Guy C.J. Abell, Lesley A. Clementson, Susan I. Blackburn, Peter D. Nichols, Anthony Koutoulis*

Heterotrophic growth of thraustochytrids has potential in co-producing a feedstock for omega-3 long-chain (≥C20) polyunsaturated fatty acids (ω3 LC-PUFA, also termed long-chain omega-3 oils) and biodiesel. Development of microalgae in Australia for biodiesel requires matching algal strains to climatic conditions and, in particular, using endemic strains to protect Australia’s unique biodiversity; biodiscovery of thraustochytrids from the southeast coast of Tasmania (temperate) and far north Queensland (tropical), Australia. A total of 36 thraustochytrid strains were isolated (19 temperate and 17 tropical) and separated into eight chemotaxonomic groups based on fatty acid and sterol composition. The eight groups clustered closely with four genera obtained by 18s rDNA molecular identification—*Aurantiochytrium*, *Schizochytrium*, *Thraustochytrium* and *Ulkenia*. Phylogenetic and chemotaxonomic groupings demonstrated similar patterns for the majority of strains. Results to date demonstrate the potential of these new Australian thraustochytrids for synthesis of other co-products, such as carotenoid pigments, sterols and other novel lipids, in addition of biodiesel and LC omega-3 oils. We provide a perspective on thraustochytrids biodiscovery from the southeast coast of Tasmania (temperate) and far north Queensland (tropical), Australia, covering a diversity of habitats including freshwater, brackish, and marine environments. In addition, different chemotaxonomic profiles from the isolation collections will be discussed. Finally, some of the major challenges faced in the biodiscovery process for novel strains of thraustochytrids will be assessed.

### 2.28. Creating a Plant-Based Sustainable Source of Essential Long Chain Omega-3 Fatty Acids—*Surinder P. Singh*

Long-chain polyunsaturated fatty acids (LC-PUFA) have a carbon backbone of at least 20 carbons in length and contain multiple double-bond desaturations. LC-PUFA can be grouped into either an omega-3 or omega-6 category based on the position of the first double-bond from the methyl (Omega) fatty acid terminus. LC-PUFA omega-3 fatty acids, like EPA and DHA, have critical roles in human health and development with studies indicating that deficiencies in these fatty acids can increase the risk or severity of cardiovascular disease, inflammatory diseases and rheumatoid arthritis, hypertension and neuropsychiatric disorders such as depression or dementia. This talk will discuss the production of EPA and DHA in plant oils. Currently, these fatty acids are predominantly sourced from fish and algal oils. Wild-harvest marine fish stocks are widely recognized to be under threat and in order to be able to meet the increasing demand for these oils there is an urgent need for an alternative and sustainable source of EPA and DHA. Oilseed plants producing high yields of EPA and DHA are, therefore, an attractive alternative to fish oils. I will describe the efforts under way in CSIRO to create an oilseed with significant amounts of EPA and DHA in its oil and how these plant oils will be able to meet the growing demand for these fatty acids.

### 2.29. *In-Vitro* Δ6-desaturase Activity: Fatty Acid and Lipid Modification of Isolated Chloroplasts from Borage Leaves and Microalgae *Isochrysis galbana—Kathrin Stähler, Siew-Young, Quek, Matthew R. Miller*

This study investigated the ability of isolated chloroplastsfrom borage (*Borago officinalis* L.) and the microalgae *Isochrysis galbana* to modify lipids *in-vitro* in order to assess Δ6-desaturase activity. Understanding the activity and the possible extraction of Δ6-desaturase may enable *in vitro* modification of oils to produce enriched *n*-6 and *n*-3 lipids.

Chloroplasts from borage leaves and *Isochrysis galbana* were incubated alone (control) or added with one of the following precursors (30 µg precursor/25 µg chlorophyll): (1) high oleic acid (C18:1*n*-9) free fatty acids (OA-FFA), (2) high α-linolenic acid (C18:3*n*-3) FFA (ALA-FFA) or (3) high ALA-monogalactosyl diacylglycerol (ALA-MGDG). Sampling took place every 30 min during the trial period and oil was extracted. Changes in fatty acid (FA) composition were determined by GC-MS and those in lipid class by Iatroscan thin layer chromatography-FID. 

We demonstrated that Δ6-desaturase activity (measured by changes in SDA/ALA ratio) was enhanced by up to 53% during incubation, when adding ALA-FFA to the microalgae chloroplasts. However, the addition of OA-FFA to the isolated chloroplasts of both species showed no significant (*P* < 0.05) changes in major FA during *in-vitro* incubation. Also, ALA-MGDG as a precursor did not improve Δ6-desaturase activity in borage and *Isochrysis galbana* chloroplasts. For borage samples, results indicated no incorporation of the added FFA to chloroplast lipids. For *Isochrysis galbana* samples, there is evidence showing the incorporation of the added FFA precursors into chloroplast glycerolipids with FFA in the ALA-FFA and OA-FFA samples decreasing from 8.4% to 2.6% and 8.4% to 2.2%, respectively after 30 min of incubation. 

Current results indicate that chloroplasts of *Isochrysis galbana* have the better ability to incorporate the added FFA precursors (OA-FFA and ALA-FFA) into chloroplast lipids and show more promising Δ6-desaturate activity compared with borage chloroplasts. 

### 2.30. Lipids of New Zealand Extremophiles—*Andrew MacKenzie, Mikhail Vyssotski, Kirill Lagutin, Jason Ryan, Kevin Lee, Xochitl, Matthew Stott*

As organisms enduring extremely harsh environment, extremophiles have, for decades, been of interest to biochemists trying to understand how the membrane integrity is preserved and metabolism maintained under conditions that would destroy other organisms instantly. Lipids have a critical role in maintaining membrane integrity, and a number of novel lipids have been discovered from extremophiles. Studies into the structures of the lipids of extremophiles are hindered by a need to establish the culture and maintain the growth of the organism outside of its natural environment in order to produce sufficient amount of biomass. 

This presentation summarizes the results of research into the discovery and structural elucidation of lipids from selected extremophiles performed at IRL during the last two years, with an emphasis on novel fatty acids and phospholipids. The phospholipids of *Chthonomonas calidirosea*, a Gram-negative, aerobic, pink-pigmented, rod-shaped bacterium with an optimal temperature for growth of 68 °C, were all found to possess a phosphatidylglyceroylalkylamine structure. Nine previously unreported phospholipids were discovered in this organism. Fatty acids of *C. calidirosea* contain about 20% of Δ-5 monoenes and 5.2% of a novel cyclopropane fatty acid—5,6-methylene hexadecanoic acid. Lipids of *Thermogemmatispora lignivorax*, a novel species of thermophilic bacteria (optimum growth temperature 50–60 °C) isolated from geothermally heated soil, were shown to contain more than 90% branched and no unsaturated fatty acids. Structures of 9 novel dimethyl branched fatty acid have been established, the major constituent of these acids, 12,17-dimethyloctadecanoic, represents 16.3% of the total fatty acid composition.

### 2.31. Variation of Major and Minor Fatty Acids in Milkfat—*Amy S. Logan and Cornelius Versteeg*

The natural fatty acid composition of milkfat differs and varies between seasons and regions. In a survey over a period of 2 years of bulk milk supplies in 1994 and 1995 from different regions in Australia large differences were observed. The literature suggests differences and variation in fatty acid composition may be caused by breed, nutrition, stage of lactation and environmental factors. The historic data were examined in more detail and attempts were made to determine some specific variations and possible causal effects. This paper will summarize the results. For example the short chain fatty acids (C4:0–C10:0) in the milkfat varied between about 9.5 and 16 wt% and the unsaturated fatty acids between 25 and 34 wt%. The average palmitic acid content increased from about 26% in predominantly early lactation to 31% in late lactation supplies. Variations in other fatty acids were much less correlated with the average stage of lactation of the supplies.

Of the minor fatty acids of nutritional interest, conjugated linoleic acid (CLA) varied between 0.38 and 1.90 wt% ; branched fatty acids C15:0 (total of anteiso- and iso-) between 0.60 and 1.09 wt%; C17:0 (anteiso-) between 0.27 and 0.61 %; and 17:0 (iso-) between 0.22 and 0.61 wt%. From a region with a relatively stable monthly milk production volume over the whole year, these variations were relatively small, for example CLA varied between 0.86 and 1.4% over the period of the survey, whereas another region with large variations in production the concentrations varied between 0.55 and 1.90 wt%. These minor fatty acids have been suggested to have anti-carcinogenic and/or atherosclerosis protective properties at levels present in milk. If substantiated, then selective consumption of milkfat containing products (e.g., whole milk, cream, butter) from particular origins will be more beneficial than from others. The levels and variations of some of the fatty acids were seasonally recurring phenomena and should therefore be predictable. However during the last 15 years farming practices have changed considerably in some regions and composition patterns could now be different, necessitating a evaluation of milks from current production systems. 

### 2.32. Astaxanthin as a Potential Antioxidant for Omega 3 Fatty Acids—*Danae Larsen, Siew-Young Quek, Laurence Eyres*

Previous research on the thermal treatment of New Zealand King Salmon revealed that the level of omega 3 fatty acid (ω-3 FA) was well preserved after various heating methods. It is hypothesized that astaxanthin, a carotenoid present in King Salmon, may play an important role in this phenomenon. Current study is therefore, aimed to evaluate the effectiveness of astaxanthin as an antioxidant to prevent lipid oxidation.

A fish oil model was designed to observe the potential of astaxanthin as antioxidant using supercritical CO_2_ extracted Salmon oil and Hoki oil. A storage trial over a period of 14 days at 60 °C was conducted in both open and closed environments. The open system used uncapped vials, whereas the closed system used nitrogen flushed capped amber vials, to house the oil samples. Astaxanthin was compared against commercial antioxidants, butylated hydroxytoluene (BHT) and alpha-tocopherol at 50 and 100 ppm. Sampling was conducted at certain time interval and the Peroxide Value (PV) and Thiobarbituric Acid Reactive Substances (TBARS) were determined according to AOCS procedures. The ω-3 FA content was analyzed by GC-FID.

The Hoki oil control (without antioxidant) had the greatest increase of PV and TBRS across the 14-day trial. In the open environment, astaxanthin effectively suppressed primary and secondary oxidation up to day 5. However, after that time period, results indicated that astaxanthin was relatively affected by the presence of light, heat and oxygen as the astaxanthin system produced relatively higher primary and secondary oxidation products. In the closed environment, astaxanthin was found to significantly (*p* < 0.05) prevent primary and secondary oxidation compared to BHT and alpha-tocopherol for 14 days. Among all the ω-3 FAs present, decosahexaenioc acid (DHA) showed the greater loss, with higher total loss in the open environment. Overall, antioxidants were found to be effectively reduced the loss of ω-3 FAs, and the 100ppm astaxanthin model system was the most effective at preventing the loss of the ω-3 FAs in the closed environment. 

### 2.33. Assessment of Oxidative Stability of Hoki Oil and its Ethyl Esters by Thermal Analysis—*Tengku R. Mohamad and John E. Birch*

The relationship between fish oil and human health has increased the consumption of fish oil and its market demand. Food manufacturers have incorporated fish oil into food products and marketed them as functional foods. However, these products are more prone to oxidation since fish oil has a high amount of polyunsaturated fatty acids. Lipid oxidation is one of the problems in the food industry as it can lower the quality of the food and reduce its shelf life. Hence physicochemical characterization of the fish oil is important before the oils can be incorporated into the food products.

Hoki is one of the commercial fish species in New Zealand and hoki oil is sold in a liquid and fish oil capsule forms. Hoki oil has also been processed as an omega-3 concentrate through ethyl ester preparation. The present study was carried out to determine the melting and crystallization characteristics of the hoki oil and its ethyl ester derivative along with their thermal and oxidative stability by Differential Scanning Calorimetry (DSC) and Thermogravimetric Analysis (TGA). The oils were supplied by the SeaDragon Marine Oil Ltd., New Zealand. DSC measurements over the temperature range −60 °C to 40 °C illustrated the melting and crystallization characteristics of the oils. Results of the thermal analysis by DSC showed that the onset time for oxidation of ethyl esters was earlier than the parent oil. A similar trend was recorded for the oxidative stability trial by TGA. The onset time for oxidation occurred earlier in the TGA than in the DSC indicating uptake of oxygen prior to decomposition. DSC and TGA can be used as a rapid method to measure oxidative stability of the oils.

### 2.34. Enzyme Activity of Fatty Acid Metabolism in Barramundi, *Lates calcarifer*: Whole-Body Mass Balance Approach—*Ramez Alhazzaa, Andrew R. Bridle, Peter D. Nichols, Chris G. Carter*

Fish oil (FO) supply is strongly dependent on the availability of wild fisheries and is under pressure from increasing demand of the competing and expanding aquaculture, agricultural, functional food and pharmaceutical industries. Some vegetable oils (VO) are rich in short-chain (SC, ≤C_18_) polyunsaturated fatty acids (PUFA) and are emerging as practical replacements for FO in aquafeeds, however, VO do not contain the *n*-3 long chain (LC, ≥C_20_) PUFA considered so beneficial to human consumers. Feeding fish on different *n*-3 precursors to increase *n*-3 LC-PUFA content in flesh is of potential interest in aquaculture. To test the metabolic mechanism of utilising dietary fatty acid (FA) and the capacity of barramundi to synthesise LC-PUFA from SC-PUFA precursors, three identical diets differing only in lipid source were fed to juvenile fish raised in freshwater and seawater for 8 weeks. Oils from *Echium plantagineum* (EO) rich in SDA (18:4*n*-3) and canola (CO) containing ALA (18:3*n*-3), were compared with a FO diet rich in EPA (20:5*n*-3) and DHA (22:6*n*-3). FA mass balance (FAMB), which involves quantification of the initial and final FA composition of the whole body and the net intake of dietary FA, showed no significant effects of different salinities on FA metabolism. Key enzymes involved in FA biosynthesis varied in apparent *in vivo* activity depending on dietary treatment. EO-fed fish, containing SDA, did not show apparent activity for the Δ-6 FA desaturase (FAD6) enzyme. CO inclusion in diets increased FAD6, which also occurred for the EO diet although to a lesser extent than when feeding on FO. Higher activity was shown by the downstream Δ5 FA desaturase (FAD5) enzyme needed to synthesise *de novo n*-3 LC-PUFA products in EO-fed fish compared to the FO and CO fed fish. FO-fed fish accumulated oleic acid and showed significantly higher apparent activity for Δ9 FA desaturase (FAD9) compared with the EO and CO treatments. Apparent β-oxidation of total dietary and *de novo* fatty acids was highest in fish fed on FO compared with VO. Barramundi tended to accumulate most of the dietary saturated (SFA) and monounsaturated FA (MUFA), but utilise PUFA for biosynthesis of LC-PUFA. FA biosynthesis did not produce high amounts of the final LC-PUFA products, in particular DHA. LC-PUFA were preferentially catabolised via peroxisomal oxidisation from the FO-diet. Using FO as the sole dietary lipid source in barramundi nutrition is a wasteful practice because barramundi is capable of accumulating considerable amounts of the ALA, SDA although retaining less EPA and DHA. VO, especially SDA-rich oils, are possible alternatives for FO in barramundi aquaculture, in terms of fish growth and health, however, the n-3 LC-PUFA that are so beneficial to human consumers are not accumulated.

### 2.35. Purification of Pollock Fish Oil using Synthetic Magnesium Silicate—*Brian S. Cooke and George E. Hicks*

Crude fish oil, such as Pollock, contains a wide variety of impurities that must be removed in order to achieve desired specifications. 

In this study, synthetic magnesium silicate was used to treat a crude Pollock fish oil sample in an attempt to remove these impurities and produce finished oil with high quality. 

Treatment of the crude Pollock oil with synthetic magnesium silicate resulted in:

- 71% Unsaponifiable matter reduction
- 84% Water reduction
- 91% Acid Value reduction
- 13% Peroxide Value reduction
- 100% Soap reduction
- 97% Color reduction
- 100% Chlorophyll removal
- 149% Improvement in oxidative stability

### 2.36. Composition and Frying Stability of *Brassica oils—Ata-ur-Rehman and Edward J. Birch*

*Brassica* (rapeseed) oils are used as culinary and frying oils and seed breeding programs have been extended from plant breeding in Canada in the 1970s to produce low erucic acid (canola) oils. Canola oil has been permitted a qualified heart health claim by the US FDA due to its unsaturated fat content. Cold pressing extracts free phenolic compounds associated with the seed fibre that would otherwise be insoluble in the alternative solvent process. High erucic *Brassica* oils contain glucosinolates that impart a sulfury odour to the oil and are considered unfit for human consumption, especially for frying.

In this study cold-pressed and solvent-extracted canola (*Brassica napus*) and a high erucic rapeseed (mustard, *Brassica juncea*) oil were analyzed for their fatty acid composition, phenolic acid content and flavor profiles by steam distillation/solvent extraction followed by GC-MS. The canola oils were subjected to frying for 50 h at 180 °C with a basket of French fries cooked for 5 min every hour. Oxidative indicators included peroxide, p-anisidine and free fatty acid values, total polar compounds, refractive index and fatty acid analyses.

Results indicated that cold pressing results in a 4 to 5-fold increase in phenolic content. High erucic oil has greater omega-3 content than canola oils. Volatiles recovered from the two types of *Brassica* oils showed an expected range of secondary oxidation products from the canola oils but were dominated by sulfur containing derivatives in the Mustard oil. Frying quality indicators appeared similar for the two canola oils during frying and the %TPC reached after 50 h was within the 24% reject limit recommended by the manufacturer. However, linoleic acid levels showed greater loss in the solvent extracted oil.

### 2.37. Oil-Water Partitioning of Naturally Derived Phenolics—*Morgane Jaffrelo, Peter A. Fagan, Claudio Ceccato and Amy S. Logan*

Lipid oxidation within an emulsion system is governed primarily by interfacial dynamics. It is proposed that a less lipophilic phenolic is more likely to locate at the emulsion interface compared with a more lipophilic compound, and therefore more effective in protecting the emulsion system to oxidative deterioration. To gain a better understanding of interfacial dynamics, a shake-flask method was used to determine the distribution coefficients (Log D) between n-octanol and a 20mM H_3_PO_4 _aqueous buffer (pH 2.5, 4.5 and 6.5) for four different phenolic components (sinapic acid, ferulic acid, caffeic acid and 4-vinyl syringol) identified in both preheated and non-preheated canola seed meal and/or oil. The concentration of each phenolic (100μM dissolved in 10µL DMSO) was measured using UV spectrometry at the respective λ_max_. Here the absorbance of the buffer containing the phenolic was measured after centrifugation at λ_max_ before (A_1_) and after (A_2_) n-octanol addition. Log D values of each phenolic components were calculated as follows: log D_pHi_ = log[(A_1_ − A_2_)/A_2_ × (V_w_/V_o_)], where V_w_ and V_o_ are the final volumes of buffer and n-octanol, respectively. Log D values were affected by pH for sinapic, ferulic and caffeic acid whereas the behaviour of 4-vinyl syringol was independent of pH. 

### 2.38. Screening Thraustochytrids for Production of Biodiesel and Long-Chain Omega-3 Oils—*Kim Jye Lee Chang, Graeme A. Dunstan, Susan I. Blackburn, Anthony Koutoulis, Peter D. Nichols*

Thraustochytrids are heterotrophic protists, commonly found in marine, brackish and other saline environments. Most thraustochytrids are characterised by the capacity to produce omega-3 long chain polyunsaturated fatty acids (ω3 LC-PUFA), including 22:6ω3 (docosahexaenoic acid, DHA) and 20:5ω3 (eicosapentaenoic acid, EPA). Previous research and development has examined their potential for synthesis of other co-products such as carotenoid pigments and sterols. Due to their fast growth rate and high lipid content, thraustochytrids have potential for producing a feedstock for ω3 oils and the shorter chain fatty acids suitable for biodiesel. In this study, we discuss the specific aspects of biodiscovery relevant to thraustochytrids including isolation from environmental samples, identification, and screening of thraustochytrids for their potential, in this case for biofuel and ω3 LC-PUFA. In addition, lipid and other constituent profiles from our recent isolation efforts will be examined. Results to date demonstrate the potential of these new Australian thraustochytrids for the production of biodiesel and LC omega-3 oils.

### 2.39. Composition, Antioxidant Capacity and Oxidative Stability of Chinese Tea Seed Oils—*Siew Young Quek, Yi Ke, Jingli Zhang*

Tea seed oil (mainly *Camellia oleifera*) has been used extensively as cooking oil in some of the southern provinces of China, such as Hunan, and has been reported to exhibit health benefits. The objectives of this study were to study the composition and antioxidative capacity of tea seed oils from southern China, as well as their oxidative stability during storage. 

The oils were obtained as refined, cold-pressed or from soxhlet extraction of tea seeds. The fatty acid composition, phytosterols and squalene were analyzed by GC-FID while the antioxidant capacity was determined by total phenolic content (Folin-Ciocalteau assay), DPPH scavenging activity and ORAC assay. Oxidative stability of tea seed oils was investigated in a storage trial at 60 °C for 12 days, in comparison to extra virgin and refined olive oils, and canola oil. Primary and secondary oxidations were measured by Peroxide Value (PV) and p-Anisidin Value (p-AV), respectively.

Results showed that oleic acid (C18:1) was the predominant fatty acid in the tea seed oils (up to 80%). Other major fatty acids identified were palmitic acid (C16:0), stearic acid (C18:0) and linoleic acid (C18:2). The tea sees oils also contained ~9% of polyunsaturated fatty acid, mainly as C18:2. Squalene and phytosterols (predominantly β-sitosterol) were present at a higher level in the crude and soxhlet extracted oils than the refined oil. The crude oil and the soxhlet extracted oil showed similar antioxidant capacities in terms of total phenolic content, DPPH scavenging acitivity and ORAC scavenging activity, which were all higher than the refined oil. Crude tea seed oil shows lower initial PV and p-AV, as well as slower onset of secondary oxidation products in comparison to the refined tea seed oil during the storage trial. Among the oil samples, canola oil showed the highest increase rate in both PV and p-AV, thus the least stability. Overall, the crude tea seed oil and extra virgin olive oil showed comparable oxidative stability. 

### 2.40. A Mechanistic Approach to Improving the Separation of Fatty Acid Derivatives Using Biscyanopropyl and Polyethylene Glycol GC Phases - *Paul M. Wynne, Bruce Fraser, Jaap DeZeeuw*

The GC analysis of polyunsaturated FAME is normally carried out using either highly polar polybiscyanopropylsiloxane or polyethylene glycol phases. The technique is effective at resolving compounds with at least two double bonds but is less effective for the resolution of mono-unsaturated FAME. Identification of mono-unsaturated long chain fatty acids is of importance for many reasons including their frequent inclusion in the human diet as margarines and vegetable oils.

Methods to achieve better chromatographic resolution of positional isomers have traditionally been based on increasing the polarity of the GC phase. Several highly polar phases based on various biscyanoalkylsiloxane chemistries have become available but the phases are typically difficult to manufacture reproducibly, exhibit poor capacity for compounds with a high degree of saturation and appear to offer no discernable separation advantage for mono-unsaturated FAME. We have studied the approach mechanistically and find that any increase in separating power provided by an increase in the phase polarity is countered by a decrease in the phase capacity. Further to this, we find that there is a mechanistic limitation to phase selectivity for compounds that are capable of specific interaction at only one point. 

We propose that using aryl rather than methyl esters with highly polar columns increases the capacity for saturated and monounsaturated fatty esters. The aryl ester provides a second site of retention that normalizes the analyte solubility in the highly polar phase and also provided a primary point of interaction with the phase from which to differentiate the number of carbons to the first point of unsaturation. The decrease in volatility relative to FAME may be overcome by selecting phenyl or perfluoroaryl esters and by utilizing the higher phase capacity with shorter columns or thinner film thickness.

### 2.41. The Stability of Bioactive Lipid Fractions Isolated from Green-Lipped Mussel *(Perna canaliculus)—Glen Marrow, Paul M. Wynne, Nicolette Kalafatis, Theo A. Macrides*

New Zealand Green-lipped mussel (*Perna canaliculus*) exhibits demonstrable anti-inflammatory activity in both in vitro and in vivo models. Our previous studies have linked activity to the relatively high concentration of free fatty acids in the species relative to other marine oils. The yield of SCF extracted oil is approximately 4% w/w to yield a fraction rich in free fatty acids and low in phospholipids. To improve oil harvest up to 9-10% w/w without loss of activity or severe regulatory implications, we have investigated ethanol extraction of lipase treated mussel that yields a free fatty acid rich fraction. GCMS is the method of choice for investigating changes in the product. 

The lipids were isolated by firstly freeze-drying the aqueous digested mussel followed by solvent extraction using ethanol. Fractions were methylated in acidic methanol and analyzed by GCMS or alternatively converted to the pyrrolidine amide derivative prior to analysis. On storage for prolonged periods, lipase pre-treated ethanol extraction of mussel tissue shows a loss of 20–60% w/w of omega-3 C20:4, C20:5, C22:5 and C22:6. Other free fatty acids are more resistant to oxidative loss and do not appear to be depleted in stored samples. Oxidation is slowed but not prevented by cold storage. 

The selectivity and magnitude of the loss of poly-unsaturated fatty acids (PUFA) during oxidation of the samples is consistent with a loss of active agents rather than with the formation of pro-inflammatory compounds. Such an analysis is entirely consistent with our previous understanding Green-lipped mussel oil in which we have demonstrated that the efficacy of the oil is linked to bioavailability of PUFA and therefore to the abundance and composition of the free fatty acid pool.

## 3. Contributors

Mahinda Y. Abeywardena. CSIRO—Food and Nutritional Sciences;

Guy C.J. Abell. CSIRO Marine and Atmospheric Research;

Kátya Abrantes. Institute of Marine and Antarctic Studies;

Ramez R. Alhazzaa. NCMCRS, University of Tasmania & CSIRO Food Futures Flagship;

Jamie G. Ayton. Australian Oils Research Laboratory;

Colin J. Barrow. Deakin University;

Katie M.M. Bauer. School of Psychology, University of South Australia;

Michelle Beresford. The New Zealand Institute for Plant & Food Research;

Will C. Bignell. Animal Production and Genetics, School of Agricultural Science/TIAR, University of Tasmania & CSIRO Food Futures Flagship;

John E. Birch. University of Otago;

Susan I. Blackburn. Energy Transformed National Research Flagship;

Jenna N. Bowyer. School of Biological Sciences;

Andrew R. Bridle. NCMCRS, University of Tasmania;

Janet Bryan. School of Psychology, University of South Australia;

Elaine J. Burgess. New Zealand Institute for Plant & Food Research;

Chris G. Carter. IMAS, University of Tasmania;

Owen J. Catchpole. Industrial Research Ltd.;

Claudio Ceccato. CSIRO Food and Nutritional Sciences;

Kim Jye Lee Chang. Energy Transformed National Research Flagship & Food Futures National Research Flagship;

Bingcan Chen. Department of Food Science, University of Massachusetts;

Chen, Z. CSIRO Preventative Health Flagship;

Lesley A. Clementson. Energy Transformed National Research Flagship;

Brian S. Cooke. The Dallas Group;

Eric A. Decker. Department of Food Science, University of Massachusetts;

Jaap DeZeeuw. Restek Corporation;

Jeff Drazen. University of Hawaii;

Graeme A. Dunstan. Energy Transformed National Research Flagship;

Peter J. Dyer. Industrial Research Ltd.;

Margret Edwards. Matiatia Grove;

Laurence Eyres. ECG Consulting;

Peter A Fagan. CSIRO Food and Nutritional Sciences;

Miriam Farrell. The New Zealand Institute for Plant & Food Research;

Bruce Fraser. Shimadzu Australasia;

Pernille Gerstenberg Kirkeby. SPX Flow Technology;

Kerrie G. Graham. Australian Oils Research Laboratory;

Guy C.J. Abell. Energy Transformed National Research Flagship;

Roger Harker. The New Zealand Institute for Plant & Food Research;

George E. Hicks. The Dallas Group;

Peter R.C. Howe. Nutritional Physiology Research Centre, University of South Australia;

Morgane Jaffrelo. CSIRO Food and Nutritional Sciences;

Nicolette Kalafatis. Natural Products Research Group, School of Medical Sciences, RMIT University;

James W. Kijas. CSIRO Livestock Industries;

Anthony Koutoulis. School of Plant Science, University of Tasmania;

Kirill Lagutin. Industrial Research Ltd.;

Danae Larsen. Food Science, School of Chemical Sciences, The University of Auckland;

Kevin Lee. Institute of Geological and Nuclear Sciences Ltd.;

Amy S. Logan. CSIRO Food and Nutritional Sciences;

Andrew D. MacKenzie. Industrial Research Ltd.;

Theo A. Macrides. Natural Products Research Group, School of Medical Sciences, RMIT University;

Rodney J. Mailer. Australian Oils Research Laboratory;

Aduli E.O. Malau-Aduli. 1Animal Production and Genetics, School of Agricultural Science/TIAR, University of Tasmania;

Maged P. Mansour. CSIRO Food Futures Flagship;

Glen Marrow. Natural Products Research Group, School of Medical Sciences, RMIT University;

Susan N. Marshall. New Zealand Institute for Plant & Food Research;

David J. McClements. Department of Food Science, University of Massachusetts;

Russel McCulloch. CSIRO Livestock Industries;

Peter L. McLennan. Graduate School of Medicine, University of Wollongong;

Matthew R. Miller. New Zealand Institute for Plant & Food Research;

Paul Miller. President Australian Olive Association;

Catherine M. Milte. School of Health Sciences, University of South Australia;

Tengku R. Mohamad. University of Otago;

Fernando Montañés. Instituto de Investigación en Ciencias de la Alimentación;

Xochitl Morgan. Institute of Geological and Nuclear Sciences Ltd.;

Karen J. Murphy. Nutritional Physiology Research Centre, University of South Australia;

Peter D, Nichols. CSIRO Food Futures Flagship & CSIRO Marine and Atmospheric Research;

Jenkins Ogwaro. Institute of Food, Nutrition and Human Health, Massey University;

Shane Olsson. The New Zealand Institute for Plant & Food Research;

Nigel J. Perry. New Zealand Institute for Plant & Food Research;

Heidi Pethybridge. Institute of Marine and Antarctic Studies;

Katrina Phillips. Institute of Marine and Antarctic Studies;

Rick Phleger. CSIRO Wealth from Oceans Flagship;

Jian G. Qin. School of Biological Sciences;

Siew-Young Quek. Food Science, School of Chemical Sciences, The University of Auckland;

Leandro M. Ravetti. Modern Olives & Australian Olive Association;

Ata-ur-Rehman. University of Otago;

Cecilia Requejo-Jackman. The New Zealand Institute for Plant & Food Research;

Jason Ryan. Industrial Research Ltd.;

Bill S. Shrapnel. Shrapnel Nutrition Consulting Pty Ltd.;

Zhiping Shen. CSIRO Food and Nutritional Sciences;

Surinder P. Singh. Food Futures National Research Flagship;

Richard P. Smullen. Ridley Aqua-Feed Pty Ltd.;

Kathrin Stähler. Food Science, School of Chemical Sciences, The University of Auckland;

Rosemary A. Stanton;

David A.J. Stone. South Australian Research and Development Institute;

Matthew Stott. Institute of Geological and Nuclear Sciences Ltd.;

Kalyana Sundram. Malaysian Palm Oil Council;

Stephen J. Tallon. Industrial Research Ltd.;

Tengku Mohamad, TR. University of Otago;

Cornelius Versteeg. CSIRO Food and Nutritional Sciences;

Patti Virtue. Institute of Marine and Antarctic Studies;

Mikhail Vyssotski. Industrial Research Ltd.;

Yi Wang. Institute of Food, Nutrition and Human Health, Massey University;

Louise R. Ward. School of Aquaculture, TAFI, University of Tasmania;

Geoff Webster. Hansells Food Group Limited;

Chakra Wijesundera. CSIRO Food Futures Flagship;

Mark Wohler. The New Zealand Institute for Plant & Food Research;

Marie Wong. Institute of Food, Nutrition and Human Health, Massey University;

Allan Woolf. The New Zealand Institute for Plant & Food Research;

Paul M. Wynne. Shimadzu Australasia;

Ke Yi. Food Science, School of Chemical Sciences, The University of Auckland;

Jock Young. CSIRO Wealth from Oceans Flagship;

Jingli Zhang. Institute for Plant and Food Research.

